# Large marine protected areas represent biodiversity now and under climate change

**DOI:** 10.1038/s41598-017-08758-5

**Published:** 2017-08-29

**Authors:** T. E. Davies, S. M. Maxwell, K. Kaschner, C. Garilao, N. C. Ban

**Affiliations:** 1 School of Environmental Studies, University of Victoria, PO Box 1700 STN CSC, Victoria, BC V8W 2Y2 Canada; 2 Department of Biological Sciences, Old Dominion University, 5115 Hampton Boulevard, Norfolk, VA 23529 USA; 3 Department of Biometry and Environmental System Analysis, Albert-Ludwigs-University, Freiburg, Germany; 4 GEOMAR Helmholtz Centre for Ocean Research Kiel, Düsternbrooker Weg 20, 24105 Kiel, Germany; 5 BirdLife International, The David Attenborough Building, Pembroke Street, Cambridge, CB2 3QZ UK

## Abstract

Large marine protected areas (>30,000 km^2^) have a high profile in marine conservation, yet their contribution to conservation is contested. Assessing the overlap of large marine protected areas with 14,172 species, we found large marine protected areas cover 4.4% of the ocean and at least some portion of the range of 83.3% of the species assessed. Of all species within large marine protected areas, 26.9% had at least 10% of their range represented, and this was projected to increase to 40.1% in 2100. Cumulative impacts were significantly higher within large marine protected areas than outside, refuting the critique that they only occur in pristine areas. We recommend future large marine protected areas be sited based on systematic conservation planning practices where possible and include areas beyond national jurisdiction, and provide five key recommendations to improve the long-term representation of all species to meet critical global policy goals (e.g., Convention on Biological Diversity’s Aichi Targets).

## Introduction

The recent increase in establishment of large marine protected areas (LMPAs), here defined as larger than 30,000km^2^ ^1^, has brought international conservation goals, including the Convention on Biological Diversity’s Aichi Target 11 and the Sustainable Development Goal 14.5, within reach^2^ but has also led to much debate about their contribution to biodiversity conservation. The contention about LMPAs stems from a lack of understanding of their contributions to biodiversity conservation. Proponents for LMPAs argue that they are essential because they can protect wide-ranging species e.g., seabirds, tunas^3, 4^, mitigate threats over larger areas or maintain pristine areas, and capture shifts associated with climatic changes^5^. Conversely, others argue that LMPAs are driven by political targets, situated in remote areas with minimal threats, and thus have limited conservation potential^1, 6, 7^.

**Figure 1:**
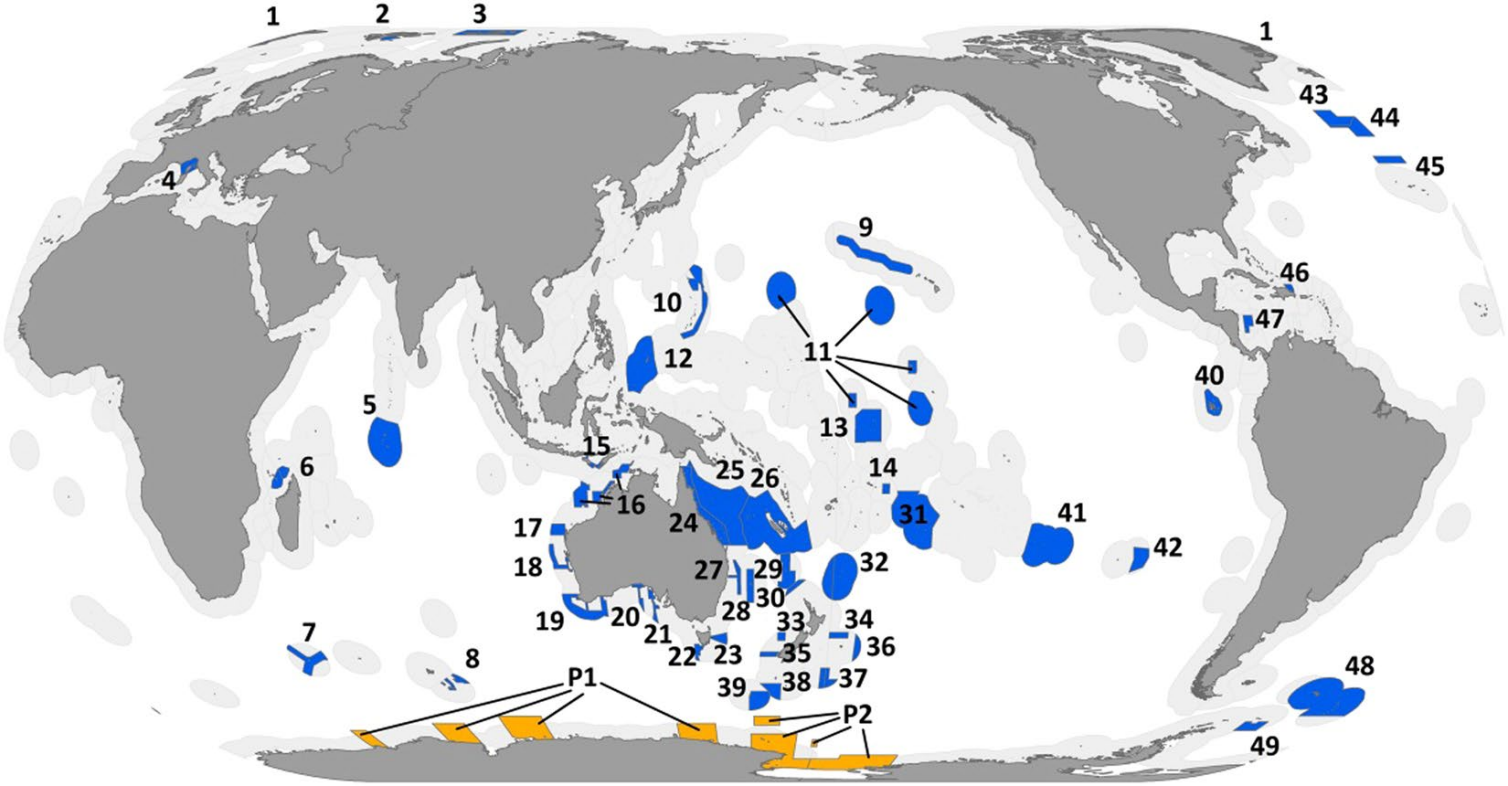
Map of all large marine protected areas used in this study. Blue areas indicate designated large marine protected areas (those with a legal boundary, as of December 2015) and are numbered; orange areas indicate proposed large marine protected areas and the number is preceded with a ‘P’ (n = 2). Full names of the large marine protected areas are in Supplementary Table 1. Figure created using ArcGIS v.10.3.1 http://desktop.arcgis.com/en/.

Despite this contention, LMPAs remain a prominent strategy for marine biodiversity conservation. In order to address this debate and move forward with large-scale marine conservation we need to understand how LMPAs contribute to representation of species. In this paper we provide the first quantification of their potential contribution to marine biodiversity conservation now and under a climate change scenario (year 2100, IPCC SRES A2 scenario)^8^ by assessing the overlap of LMPAs (Fig. 1) with all marine species for which modelled distribution data exist^9^ (n = 14,172). We use our results to identify five key recommendations to improve the long-term representation of all species and assist in meeting key global policy goals.

## Results

### Overview of species representation

The LMPAs we assessed covered 4.4% of the ocean and at least some portion of the range of 11,800 species (83% of the species assessed). Of the species within LMPAs, 26.9% had more than 10% of their range represented within LMPAs thereby meeting international conservation targets for these species, with the remaining 73.1% of species falling short of these global targets within LMPAs.

**Figure 2:**
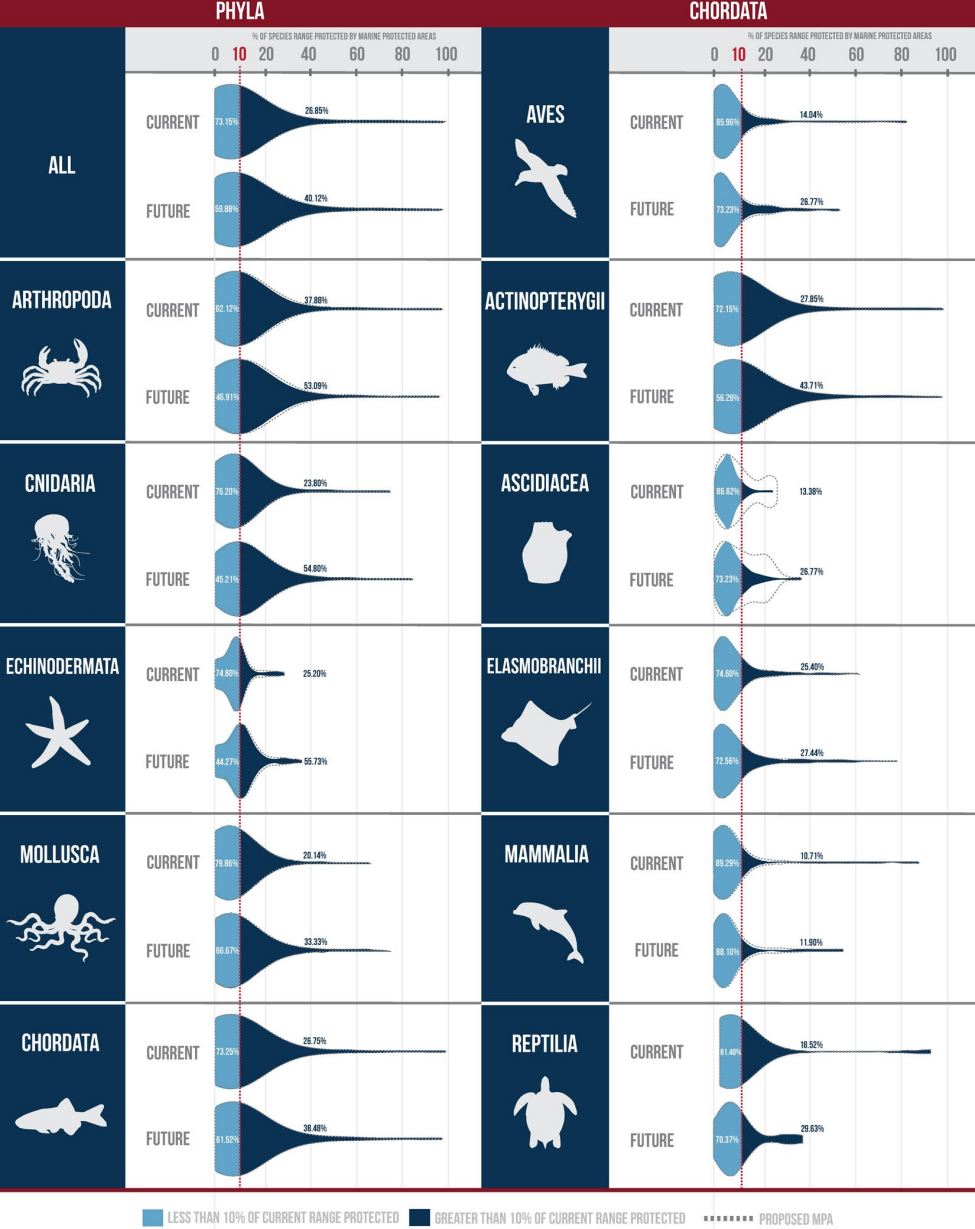
The percentage of species’ ranges represented in large marine protected areas for current and future distributions (year 2100). The violin plots show the percentage of species that do not meet (light blue), or meet or exceed (dark blue) 10% protection (red dotted line). Data are shown for all species found within designated large marine protected areas (all), for the five largest phyla (**A**); and for Chordata by its six largest classes (**B**). The light gray dashed lines indicate how these distributions would change with the addition of two networks of proposed Antarctic large marine protected areas. Figure created by Terra Communications https://terracommunications.org/.

For future distributions, LMPAs are projected to represent a higher proportion of species’ ranges in the future than today due to the projected range retractions of many species, despite this not being an explicit design intention. The percentage of species with at least 10% of their range represented in LMPAs is projected to increase to 40.1% in 2100 (Fig. 2).

Threatened species (www.iucnredlist.org) are similarly captured by LMPAs today (77.8%, n = 291) and in the future (73.5%, n = 275 in 2100). Currently 19.9% of threatened species have at least 10% of their range represented in LMPAs, with this proportion increasing to 41.5% in 2100.

The range of the majority of species (89.7%) is projected to decrease due to climate change over the next century, with a projected average range loss of 23.3%. Sharks, skates and rays (Elasmobranchii) are projected to have the largest decrease in range size, with an average loss of 39.8%, followed by birds (Aves; 29.5%), and mammals (Mammalia; 27.4%) – all higher than the average across all species (23.3%).

### Species representation by phyla

Currently, arthropods are the best represented phylum (median 8.7% of species’ range represented), and molluscs the least (median 6.1%; Fig. 2; Supplementary Table 2). Of the Chordates, ray-finned fishes (Actinopterygii) are the best represented (median 6.8%), and birds the least (median 3.6%), closely followed by mammals (median 3.7%).

The best represented phyla in 2100 are jellyfish, corals and other stingers (Cnidaria), and starfish, sea urchins, sea cucumbers (Echinodermata), both with a median of 10.4% range representation in LMPAs. Of the Chordates, the ray-finned fishes remain the best represented with an increase in their range representation (median 8.1%), and birds remain the least well represented with a slight decrease in the median range representation (3.2%; Supplementary Table 2).

**Figure 3:**
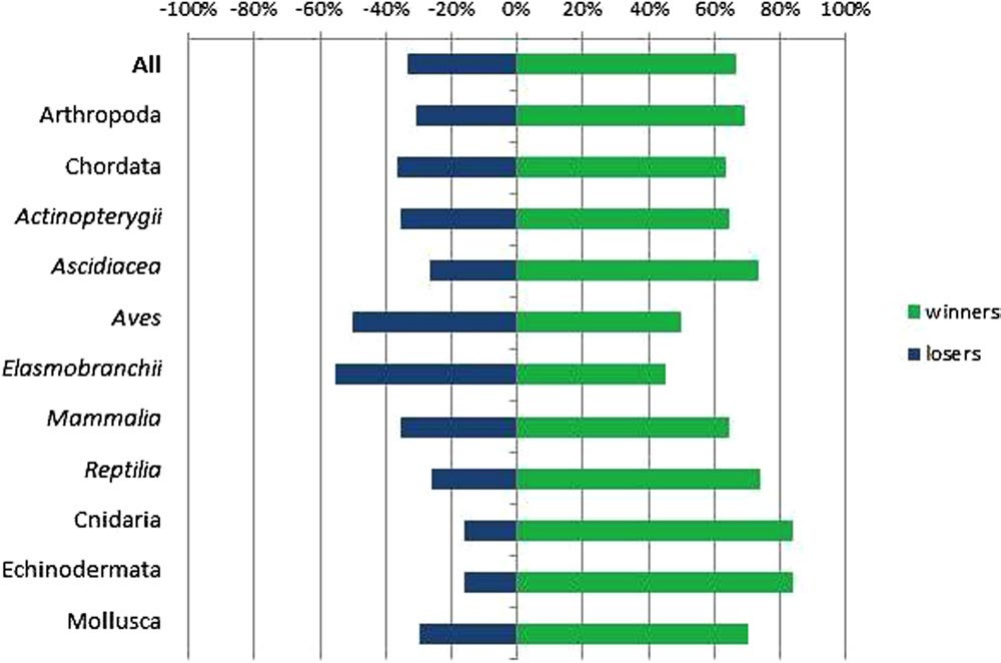
Percentage of species projected to gain (winners = green), or lose (losers = blue) representation of their range in LMPAs, under a climate change scenario by 2100. Data are shown for all species found within designated LMPAs (All), for the five largest phyla, with Chordata split by its six largest classes.

Because of these projected range retractions, the current set of LMPAs are projected to represent a higher proportion of many species ranges in the future than today, but some – sharks, skates and rays, and birds – are projected to lose protection (Fig. 3).

### Representation of wide-ranging species

Species with the largest ranges had the highest median range representation in LMPAs (>4,490,000 km^2^; median 9.31%, and 7.53% for large and extra large category respectively), with species with medium ranges (1,570,000–4,490,000 km^2^; median 3.12%) with the lowest median range representation (Supplementary Fig. 1 & Supplementary Table 3). In the future, many species are projected to experience range retractions and so the number of species within the medium range category increases, as did the median range representation of species in this category (10.5%). Species with the largest ranges generally maintain a good level of representation, with species in the large range category increasing their median representation (9.3% to 11.0%), although there was a decrease in the median representation for species in the extra large range category (7.5% to 5.5%; Supplementary Fig. 2 & Supplementary Table 3).

### Cumulative impacts in LMPAs

Overall cumulative impacts^10^ in LMPAs were significantly higher (3.11 ± 1.00) than the global average (2.43 ± 1.71; Welch’s two sample t-test: p < 0.0001, t = 5101.737; df = 20,912,753; Supplementary Table 4), indicating that LMPAs are not only located in regions with minimal threats.

## Discussion

The conservation value of large marine protected areas (>30,000 km^2^) is a source of much contention e.g., refs 1, 6 and 7 stemming from a lack of understanding about how these large areas contribute to marine biodiversity conservation. Yet despite this, large marine protected areas are increasingly being established, and now dominate areal statistics^2^. In order to address this debate and move forward with large-scale marine conservation in a productive manner, we need to understand how LMPAs contribute to the long-term representation of species. Thus, we provide the first quantification of the potential conservation value of large marine protected areas both currently and under a climate change scenario. Our empirical assessment found that LMPAs have a higher potential conservation value than might be expected by area alone. LMPAs cover 4.4% of the ocean, yet capture 83.3% of some portion of species ranges; of these, 26.9% had at least 10% of their range represented, and this was projected to increase to 40.1% in 2100. However, gaps remain, and the global set of LMPAs has not been identified in a coordinated, systematic fashion. The representation of LMPAs could be improved through using best practices in systematic conservation planning. To improve the long-term conservation benefits of LMPAs, we identify five key recommendations that should be employed to identify new LMPAs that:

### Provide protection for species currently under-represented

In contrast to many small-scale MPA networks or marine spatial planning processes, designation of LMPAs to date has been opportunistic rather than systematic, resulting in uneven representation (Fig. 2; Supplementary Table 2). To create a representative network of LMPAs - rather than the current ad hoc set - decision-support tools such as Marxan^11^ can be used to ensure representation when identifying new sites. Internationally recognized political targets, including the Sustainable Development Goal 14.5 and Aichi Target 11, call for 10% of ecologically representative marine areas to be protected by 2020. While somewhat arbitrary, such targets are useful for monitoring and evaluating global conservation efforts and are crucial in guiding conservation policies worldwide – with representation a key biodiversity conservation strategy^12^. Strategically selecting new LMPAs through a systematic approach to fill representation gaps, including increasing the representation of molluscs, birds and mammals, will increase their conservation value.

### Explicitly consider climate change

Considering the present and also projected future distributions of species in identifying new LMPAs will be critical to ensure species’ persistence. Because of projected range retractions, the current set of LMPAs is projected to represent a higher proportion of species’ ranges in the future than today, but some - sharks, skates and rays, and birds - will lose protection (Fig. 3). Coverage of sharks, skates and rays is particularly critical because they also had the largest projected decrease in range size, and these are species postulated to benefit from LMPAs^13, 14^. Identifying areas that are important to these species, such as high-use areas both now and under climate change scenarios^15–17^, will be important for targeted protection and improving the long-term representation of these species under climate change. Explicit consideration of species’ future range shifts and retractions is critical to effective representation and a number of Aichi Targets, particularly Strategic Goal C to safeguard species and Target 12 to prevent extinctions.

### Represent species with varying distributions

LMPAs had the highest representation for species with the largest ranges but species with medium ranges are not currently as well represented - highlighting additional gaps in LMPAs that can be filled with a systematic site selection approach. If LMPAs are to be beneficial for species of all ranges, explicit consideration of their representation now and in the future is necessary (Supplementary Fig. 2). Furthermore, a better understanding of the movement of individual species in relation to LMPAs will aid in designing LMPAs that will effectively capture the most important habitats. This is particularly true for highly mobile species which are purported to benefit from LMPAs. However, the benefits of LMPAs to species both with large ranges and high mobility has not yet been comprehensively assessed.

### Explicitly consider threats in priority setting processes

The mean cumulative impact score was significantly higher within LMPAs than outside (Supplementary Table 4) – refuting the critique that LMPAs protect only pristine, non-contested spaces. While protection of ‘pristine’ habitats is an important consideration^18^, future LMPAs should also be sited and managed to ensure that they reduce threats that can be spatially-mitigated such as overfishing and incidental take of vulnerable species in areas that support key life history stages or function (e.g., breeding, feeding, gestation)^19^. Reducing such key threats would enable LMPAs to be an essential element for nations to meet other Aichi targets (e.g., Target 12, threatened species; Target 6, sustainable management of fish stocks; and Target 10, minimize anthropogenic pressures on vulnerable ecosystems) and play an active role in stemming biodiversity loss.

### Move from opportunistic to systematic identification of LMPAs

Systematic identification of future LMPAs could be guided by a number of current international and regional groups or processes that employ some elements of systematic conservation planning, including the identification of Ecologically and Biologically Significant Areas under the UN Convention on Biological Diversity or several of the United Nation’s Bodies or Programs: the UNEP World Commission on Protected Areas, UN Convention on the Law of the Sea, UN Regional Seas Programme. International NGOs with capacities to analyze species distribution data can also play a role in assisting with systematic conservation planning and making these data available to support policy- and decision-making^20^. In June 2015 the UN adopted a landmark resolution that commenced negotiations toward a treaty to better manage and protect biodiversity on the high seas which comprise 64% of the world’s oceans. One potential goal is to establish a framework for implementation of MPAs on the high seas^21^. The new UN Treaty, once established, will provide an unparalleled opportunity to systematically plan LMPAs in areas beyond national jurisdiction^22^. Within the exclusive economic zones of countries, LMPAs can be placed to fill species representation gaps. Although LMPAs are subject to political realities, conservation science can help guide their placement and management to make them as effective as possible.

## Effective and equitable management of LMPAs

Representation of species ranges indicates the potential contribution LMPAs are able to make to marine biodiversity conservation. However, in order to be effective, sites need to be effectively and equitably managed. LMPAs are an important conservation strategy that have brought the 10% protection goal within reach^5^, but they are not a panacea. Complementary strategies that enhance these efforts, including smaller MPAs and appropriate fisheries management, remain critical^21, 23^. Management of LMPAs is in its infancy and data do not currently exist to assess their management effectiveness, highlighting the importance of improved monitoring and evaluation^24^. For example, global databases on MPAs have insufficient detail to classify categories of protection (e.g., whether they are no-take, or allow commercial fishing, and the spatial data of any zoning), many LMPAs lack sufficient enforcement capacities, and many IUCN listed marine species are data deficient or have not yet been assessed. Efforts to improve management are underway and should be supported, including the Big Ocean network - a peer-learning network of LMPA managers established to enhance the professional standards of practice, and long-term, effective management of large-scale marine areas^25^. Such efforts can help to share strategies to improve management effectiveness and equity, including technologies for enforcement, and stakeholder engagement^26^. In addition, prior to expansion or designation of new areas, the capacity of MPAs needs to be considered and maintained in the long-term to ensure such areas are able to deliver on the ecological and social benefits they are designed to produce^27^.

## Limitations

We used modelled species distribution data, which represent predicted relative probabilities of species occurrence (from 0.0 to 1.0) at 0.5 degree latitude-by-longitude cell resolution, and are the best data available on the distribution of marine biodiversity globally. We used a probability threshold value of 0.5 or greater to define actual species presence (or the realized niche) to improve the robustness of our findings^28^. Using a 0.5 probability reduced the chance of capturing marginal or edge habitat of species and reduced the likelihood of overestimating species range representations^29^. Previous studies have found a 0.5 threshold to be appropriate, with species distributions relatively robust across a range of thresholds^28, 30^. We determined the proportion of LMPA in each 0.5 degree cell, and because the exact distribution of species within each cell in unknown, we assumed that the area of a species range represented in LMPAs was equal to the area of LMPA coverage for grid cells where the species were present in. But where the actual species’ range is only a portion of the cell and does not overlap with the MPA the representation will be overestimated. However, we found that in 73% of the cells containing MPAs, the MPA covered more than 90% of the cell’s ocean area meaning that such overestimation is likely to be minimal. Future analyses with actual – rather than modeled – species distributions and finer resolution will be able to provide better estimates of species’ representation by LMPAs. Given the limitations of the data we used, our results can only be interpreted at the resolution of the input data, 0.5 degree cells^30^. We used one future climate scenario to model the future distributions of species, because although multiple climate scenarios exist, modeled species distribution data were only available for one scenario. We used all LMPAs that were officially designated as of December 2015 (defined as having a legal boundary; n = 53, Fig. 1 and Supplementary Table 1). We do not differentiate between varying levels of implementation, management, or enforcement because this information was not readily available. When improved data (species distributions, climate projections, management effectiveness) become available in the future, the analysis should be repeated. Additionally, as improved data becomes available it would be useful to evaluate the size and location of LMPAs for the optimal representation of biodiversity.

## Methods

We used modelled species distribution data for 14,172 marine species (28 phyla, 1256 families) derived from AquaMaps, an online species distribution modelling tool that produces standardized digital range maps of aquatic species www.aquamaps.org^9^. This is the most comprehensive and highest resolution data available on the distribution of marine biodiversity globally. The species distribution maps represent predicted relative probabilities of species occurrence at 0.5 degree latitude-by-longitude cell resolution. AquaMaps is an environmental envelope model that generates predictions about relative environmental suitability/species occurrence by relating species habitat preferences to local environmental conditions within a GIS framework. Species-specific habitat preferences with respect to five environmental parameters (depth, temperature, salinity, primary production and sea ice concentration) are derived from occurrence records available from online databases such as GBIF (www.gbif.org), supplemented by expert knowledge^29^. The subsequently calculated relative probability of occurrence of a species for each cell represents the product of probabilities for different environmental parameters based on the preferred and tolerated habitat preferences (environmental envelopes) of each species. It is assumed that the preferred range is where probability is 1, outside the range limits is where probability is 0, and between these two thresholds the relative environmental suitability decreases linearly. We used a probability threshold value of 0.5 or greater to define actual species presence (or the realized niche), as per Klein *et al*.^19^. Previous studies using AquaMaps data have found species distributions to be relatively robust across a range of thresholds^28, 30^. A higher probability of occurrence restricts species presence to species-specific areas of high environmental suitability, which corresponds to what may be considered the core range for most species, and thus a threshold of 0.5 represents a relatively conservative threshold of probability.

We used both current species distribution data and projected species distributions by the year 2100. The future species distributions are based on projected global climate conditions described under IPCC SRES A2 scenario. Predictions from this scenario are based on the assumption of independent, self-reliant countries, an increasing global population, regionally oriented economic growth, and with slow and fragmented technological change^8^.

MPAs greater than 30,000km^2^ were downloaded from MPAtlas.com^21^ and clipped to land area. Our size cut-off for large marine protected areas (LMPAs) is arbitrary; it is smaller than some^25^, and larger than others^31^, and is four magnitudes larger than the median value of MPAs globally 3.3km^2^ ^2^. We used all LMPAs that were officially designated as of December 2015 (defined as having a legal boundary; n = 53, Fig. 1 and Supplementary Table 1). We do not differentiate between varying levels of implementation or management because this information was not readily available. We consider two additional sites – East Antarctic and Ross Sea – that have been proposed for a long time (Ross Sea has since been designated (October 2016)). However, we focused our results on the designated LMPAs as of December 2015, but note the contribution of these proposed LMPAs. The MPA data were aggregated to 0.5 degree grids to match the resolution of the species range maps. We determined the proportion of the MPA in each 0.5 degree square. Because we do not know the exact distribution of species within each grid, we assumed that the area of a species range represented in MPAs was equal to the area of MPA coverage for grid cells that species were present in, and therefore we likely over-estimate species representation in LMPAs^30^. However, in 73% of cells containing MPAs, the MPA covered more than 90% of the cell’s ocean area, and in 84% of the cells containing MPAs, the MPA covered more than 50% of the cell’s ocean area (Supplementary Fig. 3). We summarize the data by the five largest phyla (Arthropoda, Chordata, Cnidaria, Echinodermata, and Mollusca), with Chordata split into its six largest classes (Actinopterygii, Aves, Ascidiacea, Elasmobranchii, Mammalia, Reptilia), because it had the largest number of species, and many of them are of conservation importance.

To assess the conservation of wide-ranging species we divided the distributional range (which is the area where species are predicted to occur, hereafter range) into four categories with an equal number of observations for the current distribution, and assessed the average representation of species by phyla in these categories for both current and future projected distributions.

We used data from Halpern *et al*.^10^, to assess the cumulative impacts within and outside of LMPAs, calculating the means and standard deviations of impact and change in impact (Supplementary Table 4).

## Electronic supplementary material

Supplementary Information

Supplementary Dataset 1
