# Supplementary material for: Large marine protected areas represent biodiversity now and under climate change
Source: Sci Rep. 2017 Aug 29;7:9569. doi: 10.1038/s41598-017-08758-5 (PMC5574922; doi:10.1038/s41598-017-08758-5)
Supplement: Supplementary file 1 — Supplementary Information [file 41598_2017_8758_MOESM1_ESM.pdf]

**Supplementary information for:**

**Large marine protected areas represent biodiversity now and under climate change.**

T.E. Davies, S.M. Maxwell, K. Kaschner, C. Garilao, N.C. Ban

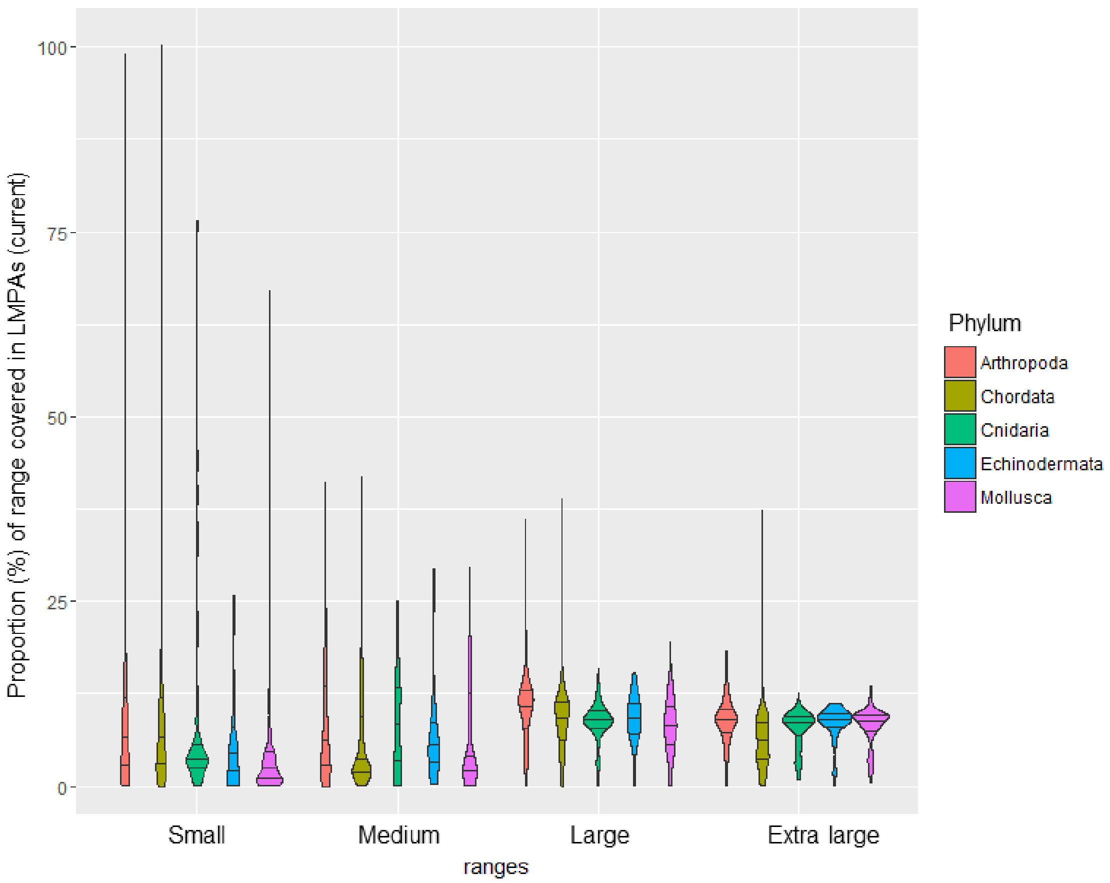


**Supplementary Fig. 1. The percentage of species range currently represented in designated LMPAs by distributional range category**: small (<1,570,000 km$^2$), medium (1,570,000 - 4,490,000 km$^2$), large (4,490,000 - 8,270,000 km$^2$), extra large (>8,270,000 km$^2$), shown for the five largest phyla. Lines within the violin plots indicate quantiles (0.25, 0.5, 0.75).

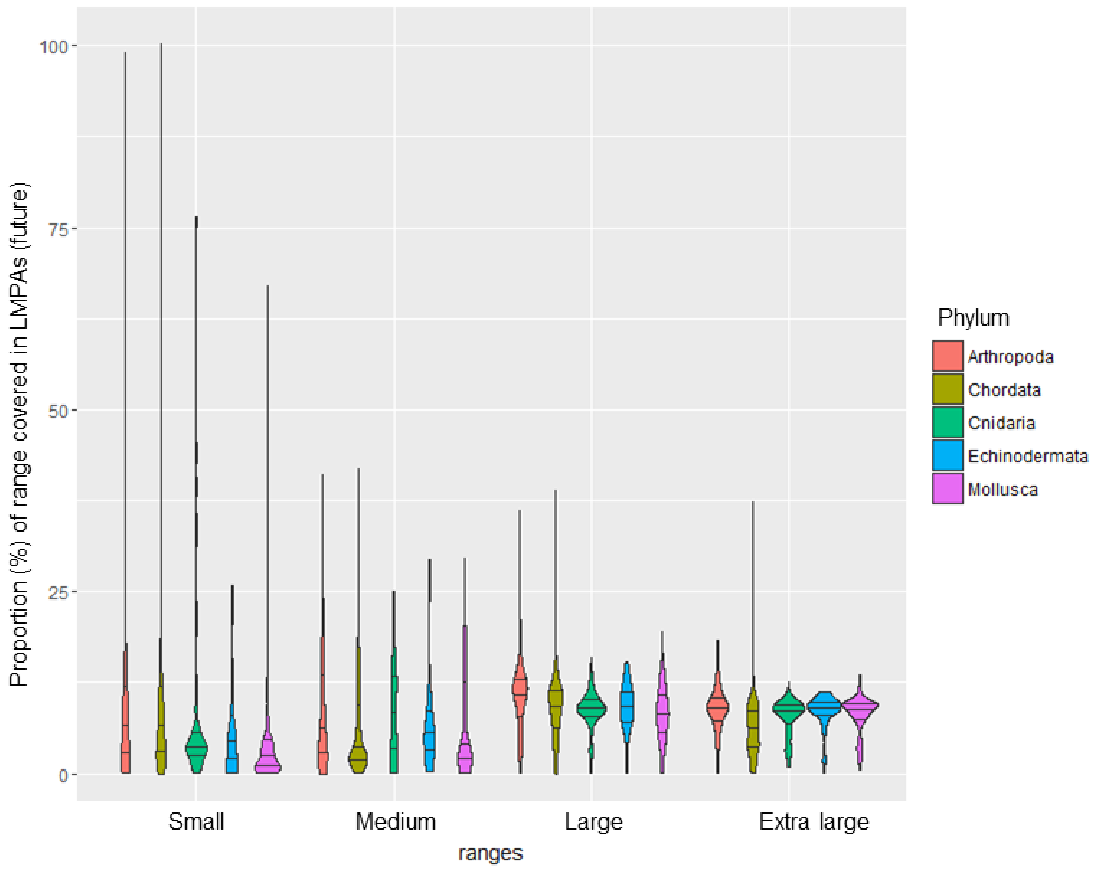


**Supplementary Fig. 2. The percentage of future species range projected to be represented in designated LMPAs in the year 2100 by distributional range category**: small (<1,570,000 $km^2$), medium (1,570,000 - 4,490,000 $km^2$), large (4,490,000 - 8,270,000 $km^2$), extra large (>8,270,000 $km^2$), shown for the five largest phyla. Lines within the violin plots indicate quantiles (0.25, 0.5, 0.75).

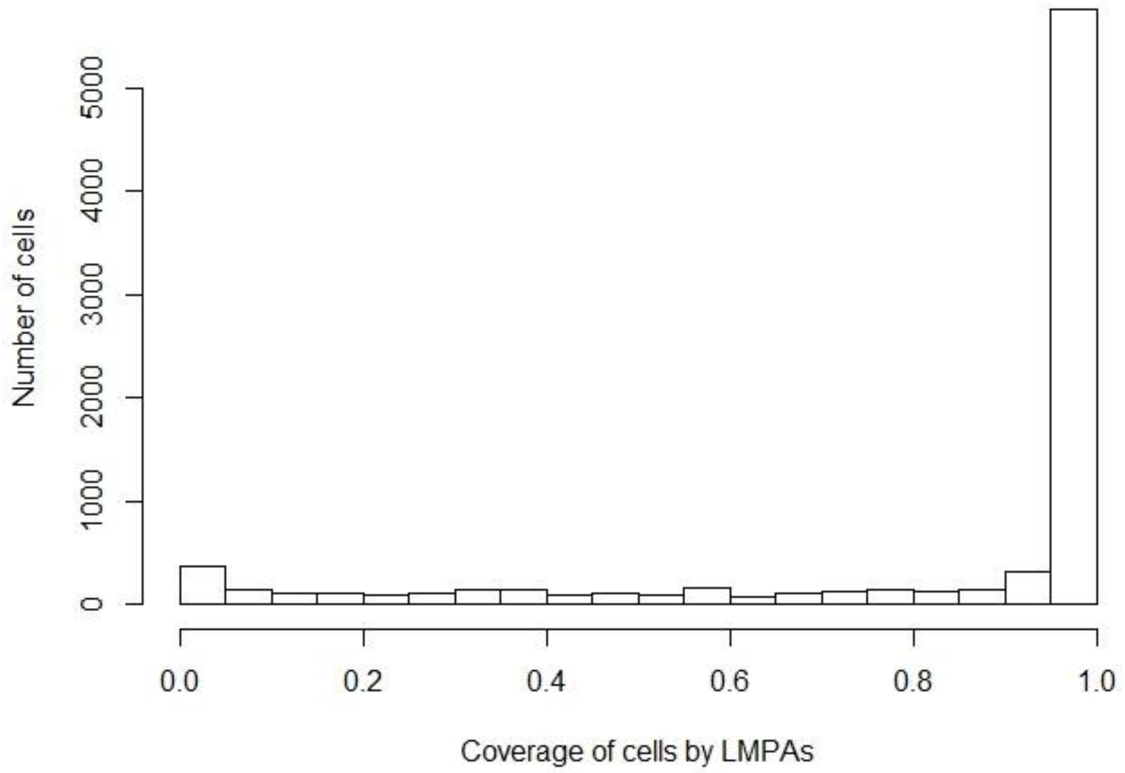


**Supplementary Fig. 3. Histogram of the number cells that contain LMPAs and the proportion of ocean area within each cell that is covered by LMPAs.**

**Supplementary Table 1.** Names of large marine protected areas used in the study (shown in Fig. 1). Designated large marine protected areas are numbered, and proposed large marine protected areas are preceded with 'P'.

| **Number** | **Name of large marine protected area** |
|---|---|
| 1 | Greenland National Park |
| 2 | Svalbard North and South Nature Reserves |
| 3 | Franz Josef Land |
| 4 | Pelagos Sanctuary |
| 5 | British Indian Ocean Territory Marine Protected Area (Chagos) |
| 6 | Gloriosos Islands & Marine Park of Mayotte |
| 7 | Prince Edward Islands Marine Protected Area |
| 8 | Heard Island and McDonald Islands Marine Reserve |
| 9 | Papahānaumokuākea Marine National Monument |
| 10 | Marianas Trench Marine National Monument |
| 11 | Pacific Remote Islands Marine National Monument |
| 12 | Palau National Marine Sanctuary |
| 13 | Phoenix Islands Protected Area |
| 14 | Rose Atoll Marine National Monument |
| 15 | Savu Sea (Tirosa Batek) Marine National Park |
| 16 | Argo-Rowley Terrace, Kimberley, & Oceanic Shoals Commonwealth Marine Reserves |
| 17 | Gascoyne Commonwealth Marine Reserve |
| 18 | Abrolhos Commonwealth Marine Reserve |
| 19 | South-west Corner Commonwealth Marine Reserve |
| 20 | Great Australian Bight Commonwealth Marine Reserve |
| 21 | Western Eyre Commonwealth Marine Reserve |
| 22 | Tasman Fracture Commonwealth Marine Reserve |
| 23 | Freycinet Commonwealth Marine Reserve |
| 24 | Great Barrier Reef Marine Park |
| 25 | Coral Sea Commonwealth Marine Reserve |
| 26 | Natural Park of the Coral Sea |
| 27 | Central Eastern Commonwealth Marine Reserve |
| 28 | Lord Howe Commonwealth Marine Reserve |
| 29 | Norfolk Commonwealth Marine Reserve |
| 30 | Norfolk Deep Benthic Protection Area |
| 31 | Marae Moana Cook Islands (Cook Islands Marine Park) |
| 32 | Kermadec Benthic Protection Area |
| 33 | Challenger South Benthic Protection Area |
| 34 | Hikurangi Deep Benthic Protection Area |
| 35 | Fiorland Transect Benthic Protection Area |
| 36 | Arrow Plateau Benthic Protection Area |
| 37 | Antipodes Transect Benthic Protection Area |

| | |
|---|---|
| 38 | Sub-Antarctic Deep Benthic Protection Area |
| 39 | Macquarie Island Commonwealth Marine Reserve |
| 40 | Galapagos Marine Reserve |
| 41 | Pitcairn Islands Marine Reserve |
| 42 | Motu Motivo Hiva Marine Park |
| 43 | Charlie-Gibbs North High Seas Marine Protected Area |
| 44 | Charlie-Gibbs South High Seas Marine Protected Area |
| 45 | Mid-Atlantic Ridge North of the Azores (MARNA) |
| 46 | Dominican Republic Marine Mammal Sanctuary |
| 47 | Seaflower Marine Protected Area |
| 48 | South Georgia and South Sandwich Islands Marine Protected Area |
| 49 | South Orkney Islands Southern Shelf Marine Protected Area |
| P1 | East Antarctic |
| P2 | Ross Sea |

**Supplementary Table 2.** Overview of representation for all marine species for both current and future scenarios in designated (and proposed) LMPAs.

| | CURRENT | | | FUTURE | | | | |
|---|---|---|---|---|---|---|---|---|
| Taxon | N (including proposed LMPAs) | Median distribution range (km$^2$) (including proposed LMPAs) | Median proportion of distribution range covered in designated LMPAs (%) (including proposed LMPAs) | N (including proposed LMPAs) | Median distribution range (km$^2$) (including proposed LMPAs) | Median proportion of distribution range covered in designated LMPAs (%) (including proposed LMPAs) | Difference in median proportion of range covered between current and future (%) | Mean loss in distributional range size by year 2100 |
| **All** | **11,800 (11,829)** | **4,588,869 (4,562,337)** | **7.0 (7.3)** | **11,611 (11,644)** | **3,292,972 (3,275,649)** | **8.3 (8.8)** | **1.3 (1.5)** | **23.8** |
| Chordata | 7,685 (7,699) | 4,154,166 (4,132,870) | 6.5 (6.7) | 7,575 (7,590) | 2,942,824 (2,934,118) | 7.6 (8.0) | 1.1 (1.3) | 25.2 |
| *Actinopterygii* | 6,696 (6,710) | 4,237,179 (4,227,795) | 6.8 (6.9) | 6,608 (6,623) | 2,974,780 (2,963,520) | 8.1 (8.3) | 1.3 (1.4) | 24 |
| *Elasmobranchii* | 441 (442) | 2,433,839 2,433,840 | 5.3 (5.4) | 427 (427) | 1,954,621 (1,954,621) | 6.0 (6.1) | 0.7 (0.7) | 39 |
| *Ascidiacea* | 269 (269) | 3,996,440 (3,996,440) | 5.6 (8.3) | 267 (267) | 3,264,213 (3,264,213) | 6.3 (10.6) | 0.7 (2.3) | 15.7 |
| *Aves* | 114 (114) | 2,685,333 (2,685,333) | 3.6 (4.0) | 111 (111) | 2,543,996 (2,543,996) | 3.2 (3.3) | -0.4 (-0.7) | 29.5 |
| *Mammalia* | 84 (84) | 28,283,718 (28,283,718) | 3.7 (4.3) | 83 (83) | 21,669,816 (21,669,816) | 4.0 (4.3) | 0.3 (0.0) | 27.4 |
| *Reptilia* | 27 (27) | 7,153,130 (7,153,130) | 7.2 (7.2) | 27 (27) | 5,712,917 (5,712,917) | 7.2 (7.2) | 0.0 (0.0) | 24.8 |
| Arthropoda | 1650 (1665) | 5,364,020 (5,322,156) | 8.7 (9.5) | 1,622 (1,639) | 3,669,198 (3,597,670) | 10.2 (11.7) | 1.5 (2.2) | 22.8 |
| Mollusca | 1380 (1380) | 4,413,540 (4,413,540) | 6.1 (6.3) | 1,333 (1,333) | 3,433,995 (3,433,995) | 7.4 (7.5) | 1.3 (1.2) | 18.7 |
| Cnidaria | 626 (626) | 6,258,470 (6,258,470) | 8.5 (8.6) | 623 (624) | 4,954,089 (4,945,435) | 10.4 (10.6) | 1.9 (2.0) | 23.4 |
| Echinodermata | 131 (131) | 6,647,560 (6,647,560) | 8.2 (8.8) | 129 (129) | 4,900,703 (4,900,703) | 10.4 (11.0) | 2.2 (2.2) | 22.6 |

**Supplementary Table 3.** The representation of marine species distributional range in designated large marine protected areas by range category: small, medium, large, extra large, for both current and projected species distributions for the five largest phyla: Arthropoda, Chordata, Cnidaria, Echinodermata, and Mollusca.

| **Distribution range (km2)** | **Distribution** | **Phyla** | **N** | **Mean (±SD) representation** | **Median representation** |
|---|---|---|---|---|---|
| **Small** (26,900-1,570,000) | Current | ***ALL*** | **2868** | **9.75 (±13.99)** | **5.04** |
| | | Arthropoda | 360 | 8.77 (±11.52) | 5.24 |
| | | Chordata | 2071 | 11.00 (±15.02) | 6.12 |
| | | Cnidaria | 83 | 6.78 (±11.17) | 3.66 |
| | | Echinoderm | 19 | 5.67 (±7.47) | 4.43 |
| | | Mollusca | 335 | 4.06 (±7.63) | 1.9 |
| | Future | ***ALL*** | **3706** | **9.57 (±14.00)** | **4.17** |
| | | Arthropoda | 459 | 8.59 (±11.32) | 4.93 |
| | | Chordata | 2676 | 10.35 (±14.74) | 4.67 |
| | | Cnidaria | 105 | 9.85 (±14.37) | 4.13 |
| | | Echinoderm | 28 | 7.97 (±10.28) | 5.78 |
| | | Mollusca | 438 | 5.63 (±11.03) | 1.32 |
| **Medium** (1,570,000 – 4,490,000) | Current | ***ALL*** | **2868** | **6.64 (±7.13)** | **3.12** |
| | | Arthropoda | 378 | 8.09 (±8.1) | 5.24 |
| | | Chordata | 1963 | 6.35 (±7.01) | 2.94 |
| | | Cnidaria | 138 | 7.92 (±2.62) | 6.58 |
| | | Echinoderm | 28 | 6.63 (±6.76) | 5.04 |
| | | Mollusca | 361 | 6.23 (±6.78) | 2.65 |
| | Future | ***ALL*** | **3356** | **11.45 (±8.81)** | **10.51** |
| | | Arthropoda | 517 | 12.52 (±8.78) | 12.16 |
| | | Chordata | 2247 | 11.10 (±8.63) | 10.21 |
| | | Cnidaria | 179 | 14.29 (±9.34) | 15.37 |
| | | Echinoderm | 34 | 11.66 (±7.63) | 12.69 |
| | | Mollusca | 379 | 10.69 (±9.44) | 7.48 |
| **Large** (4,490,000 – 8,270,000) | Current | ***ALL*** | **2868** | **8.94 (±3.86)** | **9.31** |
| | | Arthropoda | 472 | 10.29 (±4.21) | 11.04 |
| | | Chordata | 1799 | 8.78 (±3.84) | 9.24 |
| | | Cnidaria | 138 | 8.72 (±2.62) | 8.96 |
| | | Echinoderm | 36 | 8.80 (±3.62) | 9.37 |
| | | Mollusca | 327 | 8.10 (±3.73) | 8.22 |
| | Future | ***ALL*** | **2655** | **10.71 (±4.14)** | **11.02** |
| | | Arthropoda | 450 | 12.03 (±3.91) | 12.47 |
| | | Chordata | 1539 | 10.66 (±4.34) | 11.06 |
| | | Cnidaria | 274 | 10.55 (±2.94) | 10.84 |
| | | Echinoderm | 45 | 11.23 (±3.33) | 11.49 |
| | | Mollusca | 347 | 9.26 (±3.93) | 9.33 |
| **Extra large** (8,270,000-431,000,000) | Current | ***ALL*** | **2868** | **6.89 (±3.36)** | **7.53** |
| | | Arthropoda | 440 | 8.76 (±3.09) | 8.96 |
| | | Chordata | 1799 | 8.78 (±3.84) | 9.24 |
| | | Cnidaria | 171 | 7.76 (±2.50) | 8.67 |
| | | Echinoderm | 48 | 8.43 (±2.43) | 8.98 |
| | | Mollusca | 357 | 8.06 (±2.55) | 8.85 |
| | Future | ***ALL*** | **1755** | **6.21 (±3.84)** | **5.52** |
| | | Arthropoda | 224 | 8.93 (±4.24) | 9.15 |
| | | Chordata | 1223 | 5.27 (±3.53) | 4.35 |
| | | Cnidaria | 68 | 6.91 (±3.28) | 7.02 |
| | | Echinoderm | 24 | 8.61 (±3.77) | 9.81 |
| | | Mollusca | 216 | 8.27 (±2.92) | 9.13 |

**Supplementary Table 4**. Comparison of the cumulative impacts (from: *19*) within large marine protected areas and outside. The mean cumulative impact score is significantly higher within large marine protected areas (Welch's two sample t-test: $p<0.0001$, $t = 5101.737$; $df = 20,9127,53$). Total area of large marine protected areas: 15,826,452 $km^2$.

| **Cumulative impact score** | **Outside LMPAs** | **Inside LMPAs** |
|---|---|---|
| Very high (>8) | 0.01 | 0.00 |
| High (6-8) | 0.26 | 0.02 |
| Moderate (4-6) | 18.85 | 17.19 |
| Low (2-4) | 43.75 | 64.17 |
| Very low (<2) | 37.13 | 18.62 |
| **Mean (±SD)** | **2.43 (± 1.71)** | **3.11 (± 1.00)** |
